# Harnessing lignocellulosic biomass for butanol production through clostridia for sustainable waste management: recent advances and perspectives

**DOI:** 10.3389/fbioe.2023.1272429

**Published:** 2023-10-25

**Authors:** Sampathkumar Palaniswamy, Selim Ashoor, Syafira Rizqi Eskasalam, Yu-Sin Jang

**Affiliations:** ^1^ Division of Applied Life Science (BK21 Four), Department of Applied Life Chemistry, Institute of Agriculture and Life Science (IALS), Gyeongsang National University (GNU), Jinju, Republic of Korea; ^2^ Department of Agricultural Microbiology, Faculty of Agriculture, Ain Shams University, Cairo, Egypt

**Keywords:** waste management, lignocellulosic biomass, clostridia, butanol, biomass

## Abstract

The escalating waste generation rates, driven by population growth, urbanization, and consumption patterns, have made waste management a critical global concern with significant environmental, social, and economic repercussions. Among the various waste sources, lignocellulosic biomass represents a significant proportion of agricultural, agro-industrial, and municipal wastes. Biofuels are gaining attention as a promising substitute to fossil fuels, and butanol is one such biofuel that has been identified as a potential candidate due to its compatibility with existing fuel infrastructure, lower volatility, and higher energy density. Sustainable management of lignocellulosic biomass waste and its utilization in fermentation are viable alternatives to produce butanol via the promising microbial catalyst clostridia. This review provides an overview of lignocellulosic biomass waste management, focusing on recent advances in strain development for butanol production from renewable biomass with an emphasis on future perspectives.

## 1 Introduction

With the world’s population increasing, there is a concurrent increase in waste generation, prompting countries and organizations to intensify their efforts in waste reduction. Recently, particular emphasis has been placed on waste treatment to curb environmental pollution and address resource scarcity. Waste biorefinery is a promising approach in this regard. It stands at the forefront of innovation, merging cutting-edge biological conversion processes with advanced facilities to harness the immense potential of lignocellulosic biomass as a sustainable feedstock. This ingenious approach enables the generation of a diverse array of valuable products, ranging from value-added chemicals to biofuels (Sharma et al., 2019; Queneau and Han, 2022; Velvizhi et al., 2022).

Biofuels have emerged as a compelling solution, championed for their cost-effectiveness and environmentally friendly nature, especially in the face of rising petroleum prices and mounting apprehensions about the impact of fossil fuels on global warming. A wealth of studies has demonstrated remarkable yields of biofuels (ethanol and butanol) from diverse waste sources. Biobutanol has garnered significant attention due to its exceptional compatibility with combustion engines, setting it apart from bioethanol. Clostridia are known for their native butanol production and ability to utilize various substrates present in waste biomass hydrolysates (Guo et al., 2013; Du et al., 2021). However, the production of butanol by clostridia from these hydrolysates has some limitations. To overcome these limitations, the development of effective clostridia is needed.

Specifically, the metabolic engineering of clostridia holds the potential to significantly enhance the efficiency of bio-based butanol production, making it a pivotal aspect of this field. Moreover, the utilization of clostridia for butanol production from diverse feedstocks is poised to bolster the economic viability of biobutanol production. However, this mini-review does not delve deeply into the intricate details of metabolic engineering of clostridia for butanol production, as previous review articles discussed butanol production by clostridia from different feedstocks and metabolic engineering as a tool to enhance butanol production (Cheng et al., 2019; Liberato et al., 2019; Bao et al., 2020; Linger et al., 2020; Vamsi Krishna et al., 2022; Yang et al., 2022). In this mini-review, we aim to offer a forward-looking perspective while succinctly emphasizing key points, fostering a holistic comprehension of the subject. Thus, this mini-review briefly discusses recent advances in butanol production from lignocellulosic biomass waste using clostridia. We have also provided a brief overview of the application of metabolically engineered clostridial strains in the production of lignocellulosic butanol. Finally, it addresses the challenges of using clostridia for butanol production from lignocellulosic biomass waste and suggests future research directions.

## 2 Butanol production from biomass waste

The use of biomass waste is a cost-effective approach for biofuel production. By breaking down this biomass waste, several fermentable sugars like glucose and xylose are obtained, serving as substrates for the butanol production process (Cascone, 2008). Here, several biomass wastes including agricultural, agro-industrial, and municipal solid waste are discussed. Lignocellulosic biomass waste fermentations using *Clostridium* strains for butanol production are addressed in Table 1. In pursuing sustainable waste biomass utilization, it is crucial to develop high-yielding engineered strains capable of efficiently utilizing the wide range of sugars in biomass waste. This approach facilitates cost-effective butanol production using affordable carbon sources and contributes to environmental preservation by repurposing and harnessing waste materials under waste management. Utilizing waste biomass can minimize waste generation, reduce environmental impact, and promote a more sustainable approach to butanol production.

**TABLE 1:** Production of butanol from lignocellulosic biomass using clostridia.

Strain^b^	Substrate	Pretreatment	Fermentation process	Butanol titer (g/L)	Butanol yield (g/g)	References
*C. tyrobutyricum* ATCC 25755 Δ*ack* + *adhE2*-*xylTBA*	Soybean hull	Acid	Batch	15.7	0.24	Yu et al. (2015)
*C. tyrobutyricum* ATCC 25755 Δ*ack* + *adhE2*	Cassava baggase	Hydrothermal	Batch	15.0	0.30	Huang et al. (2019)
*C. tyrobutyricum* ATCC 25755 (KCTC5387) Δ*cat1*::*adhE2*	Paper mill sludge	-	SHF	16.5	0.26	Cao et al. (2020)
*C. cellulovorans* DSM 743B Δ*araR* Δ*xylR* + *ter*-*adhE1-CAT1*-*xylT*	Corn cobs	Alkali	CBP	4.96	-	Wen et al. (2020)
*C. cellulovorans* DSM 743B Δspo0A*11 + *adc*-*ctfAB*-*adhE1*-*spo0A*	Corn cobs	Alkali	CBP	3.47	-	Wen et al. (2019)
*C. tyrobutyricum* ATCC 25755 Δ*ack* + *adhE2*	Sugarcane bagasse	Acid	Batch	11.76	0.22	Li et al. (2019)
*C. tyrobutyricum* ATCC 25755 Δ*ack* + *adhE2*	Soybean hull	Acid	Batch	14.0	0.29	Li et al. (2019)
*C. tyrobutyricum* ATCC 25755 Δ*ack* + *adhE2*	Cotton stalk	Acid	Batch	15.8	0.31	Li et al. (2019)
*C. acetobutylicum* L7 + *GlcG*	Corn stover	Acid and Alkali	SSF	10.8	0.18	Wu et al. (2021)
*C. acetobutylicum* L7 + *GlcG*	Corn stover	Acid	Batch	10.0	0.22	Wu et al. (2019)
*C. acetobutylicum* MTCC 481	Rice straw	Acid	Batch	12.7	0.38	Ranjan et al. (2013)
*C. acetobutylicum* ABE 1201	Corn cob bagasse	Alkali	Batch	9.4	0.13	Cai et al. (2016)
*C. cellulovorans* ATCC 35296 and *C. beijerinckii* 10132	Wheat straw	Biological	CBP	14.2	-	Valdez-Vazquez et al. (2015)
*C. beijerinckii* NCIMB 8052	Rice straw	Alkali	Two-stage fermentation	15.9	0.47	Chi et al. (2018)
*C. saccharoperbutylacetonicum* N1-4	Switchgrass	Acid	SSF	8.6	0.16	Wang et al. (2019)
*C. acetobutylicum* GX01	Sugarcane bagasse	Alkali	Batch	14.17	0.22	Pang et al. (2016)
*C. beijerinckii* NRRL B-466	Apple pomace ultrafiltration sludge	Acid	Batch	9.3	0.24	Maiti et al. (2016)
*C. saccharoperbutylacetonicum* N1-4 (ATCC 13564)	Palm oil mill effluents sludge	-	Batch	10.35	0.29	Hipolito et al. (2008)
*Clostridium* sp. AS3	Cassava waste residue	Acid	Batch	8.01	0.25	Johnravindar et al. (2021)
*C. acetobutylicum* NJ4 and *Thermoanaerobacterium thermosaccharolyticum* M5	Corn cobs	-	CBP	7.61	-	Jiang et al. (2020)
*C. acetobutylicum* NRRL B-591	Organic fraction of municipal solid waste	Organosolv	Batch	8.57	-	Farmanbordar et al. (2018)
*C. acetobutylicum* NRRL B-591 and *Mucor indicus* 22424 CCUG	Organic fraction of municipal solid waste	Organosolv	Batch	7.9	-	Ebrahimian et al. (2022)
*C. acetobutylicum* DSM 1731	Domestic organic waste	Mansonite steam explosion	Batch	1.5^a^	-	Claassen et al. (2000)
*C. beijerinckii* B-592	Domestic organic waste	Mansonite steam explosion	Batch	0.9^a^	-	Claassen et al. (2000)

^a^ ABE titer

^b^ Δ gene deletion or inactivation, + gene overexpression.

### 2.1 Agricultural waste

Agricultural wastes refer to the materials produced at different stages of the agricultural process, including final products, by-products, and raw materials, that are no longer useful and are usually discarded (Ylä-Mella et al., 2022). To tackle the environmental challenges posed by agricultural waste, it is essential to adopt eco-friendly strategies that promote waste reduction and recycling. Instead of simply discarding these materials, they can be valorized and transformed into valuable resources through waste conversion processes. This helps create a more sustainable and circular economy and contributes to socioeconomic development, energy security, and resource conservation. By adopting these strategies, we can effectively manage agricultural waste and promote a cleaner and more prosperous future (Chilakamarry et al., 2022). Growing interest is seen nowadays in producing butanol sustainably using agricultural residues. This is in response to concerns about the competition between fuel and food production, which could potentially drive up food prices (Qureshi et al., 2014).

Waste generated from agricultural activities comprises cellulose, hemicellulose, lignin, ash, and protein extractives, which are complex molecular structures of lignocellulosic biomass. Microorganisms can degrade these waste materials into simple monomers, providing a potential renewable energy source (Ge et al., 2021). The feedstocks mainly comprise agricultural residues and crop wastes, including rice straw, wheat straw, corn cobs, and rice husk.

Numerous research groups have recently shown how biobutanol is produced from agricultural wastes (Marchal et al., 1992; Kapoor et al., 2020; Mujtaba et al., 2023). Rice straw was employed in several studies for biobutanol production (Moradi et al., 2013; Amiri et al., 2014). The bacterial strain *C. acetobutylicum* MTCC 481 produced 12.7 g/L of butanol from rice straw (Ranjan et al., 2013). In another study, prior to acetone-butanol-ethanol (ABE) fermentation, corn cob bagasse (CCB) was pretreated with a NaOH solution to remove lignin and improve cellulase accessibility. The bacterial strain *C. acetobutylicum* ABE 1201 could produce 9.4 g/L of butanol from CCB (Cai et al., 2016). In a study on biological treatment, lignin, hemicellulose, and amorphous cellulose levels were reduced, increasing wheat straw fermentability to produce butanol. The treated wheat straw was fermented with a coculture of *C. cellulovorans* 35296 and *C. beijerinckii* 10132, resulting in butanol production of 14.2 g/L (Valdez-Vazquez et al., 2015). Wang et al. (2019) used the pretreated switchgrass in simultaneous saccharification and fermentation to produce butanol by *C. saccharoperbutylacetonicum* N1-4, yielding 8.6 g/L butanol.

### 2.2 Agro-industrial waste

Agro-industrial wastes are generated every year in large quantities. Only 20% of agro-industrial food waste is being repurposed for animal feed, while the remaining amount is either landfilled, incinerated, or composted. Pang et al. (2016) employed the strain *C. acetobutylicum* GX01 to produce butanol from alkali-pretreated sugarcane bagasse. The butanol production was 14.17 g/L. Maiti et al. (2016) obtained butanol production of 9.3 g/L from apple pomace ultrafiltration sludge using the bacterial strain *C. beijerinckii* NRRL B-466. *Clostridium* sp. AS3 achieved butanol production of 8.1 g/L using cassava waste residue hydrolysate (Johnravindar et al., 2021).

Surplus starchy grains and agro-industrial process waste effluents are frequently used as fermentation feedstock due to their affordability. Using better farming practices, Southeast Asia’s oil and sago palms have been converted into sustainable bioresources. Palm oil mill effluents (POME) and crude palm oil (CPO) are products of the industrial processing of oil palm, with CPO being an essential commodity in the international vegetable oil market. POME disposal is still a problem. However, bioconversion has been considered as a potential pollution-control measure. In nations that produce palm oil, like Malaysia, where 15.2 million tons of POME are produced annually, POME is aimed for ABE fermentation which has the potential to be a cheap substrate (Al-Shorgani et al., 2015). In the ABE fermentation of *C. saccharoperbutylacetonicum* N1-4 (ATCC 13564), the growth and butanol production were shown to be supported by the hydrolysate of the separator sludge from POME. However, the lack of fermentable sugars in separator sludge hydrolysate contributes to the low product levels. *C. saccharoperbutylacetonicum* N1-4 (ATCC 13564) produced 10.35 g/L of butanol using separator sludge hydrolysate from POME and sago starch hydrolysate without the need for any extra nutrient supplementation (Hipolito et al., 2008).

### 2.3 Municipal solid waste

Municipal solid waste (MSW) is a heterogeneous blend of non-biomass combustible materials, plant and animal products, and other garbage. It can be used as a renewable biomass source to make chemicals and fuels (Vuppaladadiyam et al., 2022). Using municipal solid waste instead of gasoline might drastically reduce greenhouse gas emissions by between 29.2% and 86.1% (Shi et al., 2009).

From the biodegradable portion of MSW treated with ethanol organosolv, the strain *C. acetobutylicum* NRRL B-591 generated 8.57 g/L of butanol (Farmanbordar et al., 2018). To completely use the energy potential of the MSW organic fraction, co-cultivation of the fungal strain *Mucor indicus* 22424 CCUG with *C. acetobutylicum* NRRL B-591 was performed, yielding 7.9 g/L of butanol (Ebrahimian et al., 2022). Domestic organic waste (DOW) has been the subject of numerous studies looking at it as a substrate for butanol production (Kartik et al., 2022). ABE was generated from DOW hydrolysate by *C. beijerinckii* B-592 and *C. acetobutylicum* DSM 1731 at rates of 0.9 and 1.5 g/L, respectively (Claassen et al., 2000).

## 3 Bioprocess to overcome the complicated nature of feedstocks

Various fermentation techniques have been used after pretreatment to overcome the challenges posed by the complicated nature of feedstocks and feedback inhibition in the production of waste lignocellulosic butanol. These techniques include separate hydrolysis and fermentation (SHF), simultaneous saccharification and fermentation (SSF), simultaneous saccharification and co-fermentation (SSCF), and consolidated bioprocessing (CBP) (Parisutham et al., 2014; Haldar and Purkait, 2020). By developing strains with maximum butanol yield and ensuring efficient utilization of sugars from waste biomass, integrating the bioprocess with these advancements unlocks their full potential for waste management. In SSF, enzymes break down lignocellulosic material waste into simple sugars, and then butanol is produced using those sugars. Saccharification is carried out simultaneously with fermentation, reducing production cost and overall time (Islam et al., 2021). However, one major limitation of SSF is that the sugars released during hydrolysis can inhibit the activity of the cellulase enzymes, which are responsible for breaking down the lignocellulosic waste. This reduces butanol yield (Haldar and Purkait, 2020). In the SSF process, it is hard to achieve high cellulase activity, total sugar yield, and butanol generation with this temperature matching (He et al., 2017; Li et al., 2018).

A new process with a modification of SSF resulted in simultaneous co-saccharification and fermentation (SCSF). It is an alternative to increase the cellulosic butanol production in *C. acetobutylicum* using a soluble oligomer and regenerated cellulose (Seifollahi and Amiri, 2020). Continuous ABE fermentation has been made possible to meet expectations using a continuous bioreactor that employs immobilized cells and multi-stage fermentation SCSF. In continuous fermentation, solvent concentrations and productivity are increased because two-stage and multi-stage fermentation systems absorb acids, convert them efficiently into solvents, and utilize the available substrates completely (Chang et al., 2016). Using alkaline-pretreated rice straw, a novel two-stage fermentation process was developed to maximize sugar utilization. Butanol production of 15.9 g/L was attained using the bacterial strain *C. beijerinckii* NCIMB 8052 (Chi et al., 2018).

Consolidated bioprocessing (CBP) is created by employing a single microbe or microbial consortium in a single continuous process through the fermentation of a mixture of sugars obtained after cellulase synthesis and lignocellulose hydrolysis (Olson et al., 2012; Jiang et al., 2019). Waste lignocellulosic butanol fermentation has been subjected to several CBP processes, either through the genetic modification of strains (Lin et al., 2015; Bao et al., 2019;Tian et al., 2019) or by co-cultivating saccharolytic strains like *C. cellulolyticum* (Salimi and Mahadevan, 2013) and *C. thermocellum* (Nakayama et al., 2011) for biofuel production. A microbial consortium of *Thermoanaerobacterium thermosaccharolyticum* M5 and *C. acetobutylicum* NJ4 could produce butanol from untreated corn cobs through CBP. The butanol production was 7.61 g/L (Jiang et al., 2020).

## 4 Clostridia development strategies to overcome the challenges of butanol production from lignocellulosic biomass waste

**FIGURE 1:**
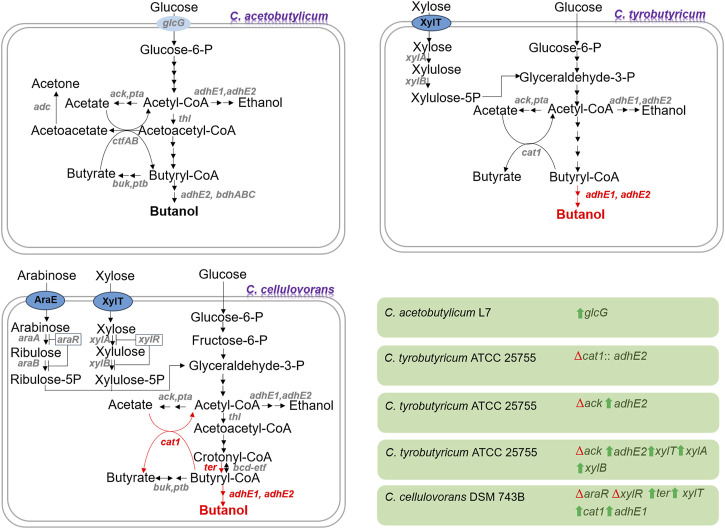
Some notable instances of metabolically engineered clostridial strains employed in the production of lignocellulosic butanol. Pathways shown in red color are absent in the wild-type strain. The red triangle and green arrow indicate gene deletion (inactivation) and overexpression, respectively. The genes are displayed in italics, and their corresponding enzymes are as follows: *glcG*: enzyme II of phosphotransferase system, *cat1*: CoA transferase, *adhE1* and *adhE2*: bifunctional aldehyde/alcohol dehydrogenase, *ack*: acetate kinase, *pta*: phosphotransacetylase, x*ylT*: xylose proton symporter, *xylB*: xylulokinase, *xylA*: xylose acetate kinase, *xylR*: xylose utilization negative regulator, *araE*: arabinose proton symporter*, araR*: arabinose utilization negative regulator, *araA*: arabinose isomerase, *araB*: ribulokinase, *thl*: thiolase, *ter*: trans-enoyl-coenzyme A reductase, *adc*: acetoacetate decarboxylase, *buk*: butyrate kinase, *ptb*: phosphotransbutyrylase, *ctfAB*: CoA transferase, *bcd*: butyryl-CoA dehydrogenase, *etf*: electron transfer flavoprotein, and *bdhABC*: NADPH-dependent butanol dehydrogenase.

Based on advances in metabolic engineering and synthetic biology tools, strain development in butanol production is improved. Strain development has been accelerated for butanol production by developing tolerance to toxic compounds, improving substrate utilization and butanol selectivity and productivity in lignocellulosic biomass waste (Birgen et al., 2019). There are different cases of strain developments considering waste biomass treatment and bioprocessing (Figure 1).

Different physical, chemical, and biological approaches are devised for detoxifying harmful inhibitors. However, these methods still face challenges in generating wastewater, raising energy expenses, and losing sugar, which hinders their economic and environmental feasibility at the industrial level (Xue et al., 2017). Mutagenesis, genetic engineering, and metabolic alterations create inhibitor-resistant strains capable of carrying out butanol fermentation from waste lignocellulosic materials, which eliminates the need for detoxification processes (Cho et al., 2019). One such example is the overexpression of the glucose-specific phosphotransferase system (*glcG*) gene in the bacterial strain *C. acetobutylicum* L7, which enabled the strain to utilize the hydrolysate of corn stover without detoxification and produce 10 g/L butanol. This achievement represents a substantial increase of 300% and 400% in butanol production compared to the control and *glcG*-lacking strains, respectively (Wu et al., 2019). To test the thermotolerance of the engineered strain, it was employed in SSF at 42 °C for butanol production using corn stover pretreated with H_2_SO_4_ and NH_4_OH. It could grow and produce 10.8 g/L of butanol under these conditions (Wu et al., 2021).

Different strategies were employed to modify the bacterium *C. cellulovorans* DSM 743B to enhance butanol production. It was engineered via integrated metabolic and evolutionary engineering by overexpressing the alcohol/aldehyde dehydrogenase (*adhE1*), CoA transferase (*ctfAB*), and acetoacetate decarboxylase (*adc*) genes from *C. acetobutylicum* ATCC 824, overexpressing the sporulation regulator (*spo0A*) gene, and eliminating the sporulation regulator (spo0A*11) gene along with adaptive laboratory evolution. This approach resulted in butanol production of 3.47 g/L from alkali-extracted corn cobs (Wen et al., 2019). In another study, the CoA transferase (*cat1*) gene from *C. tyrobutyricum* DSM 2637, and trans-enoyl-coenzyme A reductase (*ter*) gene from *Treponema denticola* were overexpressed in *C. cellulovorans* DSM 743B-*adhE1*. Also, the xylose metabolism was engineered by inactivating *araR* (*Clocel_1253*) and *xylR* (*Clocel_0594*) along with overexpressing *xylT* (*CA_C1345*). The resulting strain could produce 4.96 g/L of butanol from alkali-extracted corn cobs (Wen et al., 2020).

The bacterium *C. tyrobutyricum* ATCC 25755 was engineered by overexpressing the aldehyde/alcohol dehydrogenase 2 (*adhE2*) gene from *C. acetobutylicum* ATCC 824 and inactivating the acetate kinase (*ack*) gene to improve butanol production. The engineered strain could produce 10 g/L of butanol from glucose (Yu et al., 2011). The performance of this engineered strain was better when it was employed for butanol production from the hydrolysates of cotton stalk, soybean hull, and sugarcane bagasse, as the butanol titers were 15.8, 14.0, and 11.76 g/L, respectively (Li et al., 2019). Also, its performance was better when it was used for butanol production from the hydrolysate of cassava baggase, as it produced about 15.0 g/L of butanol (Huang et al., 2019). To overcome the glucose catabolite repression and improve xylose utilization, the xylose metabolism genes *xylTBA* (*xylT, xylA,* and *xylB*) from *C. acetobutylicum* ATCC 824 were co-overexpressed along with the *adhE2* gene in *C. tyrobutyricum* ATCC 25755 (∆*ack*). The resulting strain could utilize glucose and xylose present in the hydrolysate of soybean hull and produce 15.7 g/L butanol (Yu et al., 2015). Zhang et al. (2018) replaced the butyryl-CoA/acetate CoA transferase (*cat1*) gene with the *adhE2* gene in *C. tyrobutyricum* ATCC 25755 (KCTC5387). The engineered strain was used for butanol production from the hydrolysate of paper mill sludge supplemented with corn steep liquor, leading to butanol production of 16.5 g/L (Cao et al., 2020).

## 5 Conclusion and perspectives

Lignocellulosic biomass is a promising feedstock for sustainable butanol production. Clostridia are known for their native butanol production and ability to utilize various substrates present in lignocellulosic biomass hydrolysates (Guo et al., 2013; Du et al., 2021). As a result, several research studies have focused on enhancing the performance of clostridial strains for butanol production and harnessing lignocellulosic biomass waste. The enhanced strains by metabolic engineering have only seen limited application in the context of lignocellulosic biomass utilization for butanol production. While they have primarily been utilized with pure glucose for butanol production (Formanek et al., 1997; Nair et al., 1999; Harris et al., 2000; Harris et al., 2001; Tomas et al., 2003; Jang et al., 2012; Xu et al., 2015; Du et al., 2021; Jang et al., 2023). Several engineered strains are yet to be harnessed for butanol production using lignocellulosic biomass waste. The convergence of these research strands is poised to expedite the development of economically feasible butanol production technologies.

On the other hand, the production of butanol by clostridia from hydrolysates of lignocellulosic biomass has some limitations, including strain inability to tolerate inhibitors present in biomass hydrolysates, strain intolerance to high concentrations of butanol, and low titer. To overcome these limitations, further engineering of clostridia is needed to enhance strain tolerance to inhibitors and butanol, increase strain efficiency in metabolizing the wide range of sugars present in biomass hydrolysates, and redirect the carbon flow toward butanol synthesis for attaining the maximum butanol production capacity from lignocellulosic biomass waste. Identification of the cheapest carbon sources through the exploration of waste lignocellulosic biomass and its pretreatment methods is required. The fermentation process should also be optimized. Combining these areas to produce butanol from the cheapest carbon source with sustainable waste biomass could result in the maximum utilization of waste resources towards effective waste management with the fullest productivity.
